# Supplementary material for: HLA-DRB1 allele and autoantibody profiles in Japanese patients with inclusion body myositis
Source: PLoS One. 2020 Aug 18;15(8):e0237890. doi: 10.1371/journal.pone.0237890 (PMC7437458; doi:10.1371/journal.pone.0237890)
Supplement: S1 Table — (DOCX) [file pone.0237890.s001.docx]

TableS1

The whole dataset of 83 Japanese patients with inclusion body myositis (IBM) subjected to the present analysis.

01:01: DRB1*01:01; 04:10: DRB1*04:10; 15:02: DRB1*15:02; cN1A: Anti-cN1A antibodies; HCV: Infection of hepatitis type C virus; AA: Autoantibodies ; SE: Statin exposure; C: Cancer; SAD: Systemic autoimmune disease; SLMW: Severe leg muscle weakness; FFW: Finger flexion weakness; FMI: Facial muscle involvement; NMW: Neck muscle weakness; CMI: Cardiac muscle involvement; RMI: Respiratory muscle involvement; MA: Muscle atrophy; M: Myalgia; A: Arthropathy; RP: Raynaud phenomentn; ILD: Interstitial lung disease; CK: Creatine kinase; ECRP: Elevated C-reactive protein; ANA: ; Antinuclear antibody positivity
