# Supplementary material for: HLA-DRB1 allele and autoantibody profiles in Japanese patients with inclusion body myositis
Source: PLoS One. 2020 Aug 18;15(8):e0237890. doi: 10.1371/journal.pone.0237890 (PMC7437458; doi:10.1371/journal.pone.0237890)
Supplement: S7 Table — (DOCX) [file pone.0237890.s007.docx]

Table S7

Differences in clinical features and DRB1 alleles between the IBM patients with both HCV infection and anti-cN1A antibodies and others.
