# Supplementary material for: HLA-DRB1 allele and autoantibody profiles in Japanese patients with inclusion body myositis
Source: PLoS One. 2020 Aug 18;15(8):e0237890. doi: 10.1371/journal.pone.0237890 (PMC7437458; doi:10.1371/journal.pone.0237890)
Supplement: Attachment — Submitted filename: Response to Reviewers.docx [file pone.0237890.s008.docx]

Reviewer #1

The manuscript is clearly written and well-organized. The authors describe a study of 83 IBM patients and 460 Japanese matched-controls. High-resolution HLA DRB1 sequencing revealed multiple alleles significantly associated with IBM. Also, certain clinical signs and symptoms were likewise associated with disease-susceptibility alleles. Additional analyses of autoantibody profiles and Hepatitis C status revealed some non-significant although interesting trends. I recommend the manuscript for publication in PLOS ONE without further comment.

We appreciate the kindly comment.

Reviewer #2

Many thanks for asking me to review this HLA study of patients with IBM in a Japanese population. The findings are of some interest and confirm an association of HLA-DRB*01:01, which has previously been confirmed in other ethnic populations in IBM. The manuscript is well written with excellent English and no obvious grammatical errors - the authors should be commended for this.

1. The study is somewhat small in number, and the fundamental issue with the methodology is that no attempt has been made to correct for multiple comparisons. Paragraph in table 1, there are 29 alleles therefore, if a Bonferroni correction for multiple comparisons is applied the P value must be multiplied by 29. The DRB1*01:01 and DRB1*04:10 associations do persist after this correction. Therefore, it is more than likely that given the lack of statistical power, the described HLA/clinical associations analyzed are likely by chance and again a correction for multiple comparisons must be applied here. Any associations with clinical features or serology described are at best speculative.

As suggested, we performed the statistical analyses with a Bonferroni correction for multiple comparisons. We added the following sentences.

Page 6

“Bonferroni-corrected p values were obtained by multiplying the observed p values by the number of DRB1 alleles (× 29).”

Page 9

“Further analyses using Bonferroni-corrected p value also revealed that these alleles showed a tight association with Japanese IBM patients.”

“Among these alleles, *DRB1*09:01* showed still significant after a Bonferroni correction for multiple comparisons. In fact, *DRB1*09:01* was only found in one IBM patient, although it was a relatively common allele in the Japanese population.”

Page 12

“(ii) there was a weak association between the *DRB1*01:01* allele and clinical features (severe leg muscle weakness and muscle atrophy);”

2. At the end of the study discussion, there is no research agenda or suggestions for further work based on the findings. Surely an ethnographic study comparing Caucasian and Japanese patients would be of some interest here.

As suggested, we modified the final sentences.

Page 14

“It is possible that information of regarding HLA or other genetic risk factors will be useful for predicting a favorable response to immunotherapies or other therapies for IBM patients. In the future, an ethnographic study comparing Caucasian and Asian patients would give us important information.”

3. I don't feel that the Venn diagram in figure 1 is necessary and adds anything to the manuscript.

We appreciate the thoughtful suggestion. We deleted the figure 1.
