# Supplementary material for: HLA-DRB1 allele and autoantibody profiles in Japanese patients with inclusion body myositis
Source: PLoS One. 2020 Aug 18;15(8):e0237890. doi: 10.1371/journal.pone.0237890 (PMC7437458; doi:10.1371/journal.pone.0237890)
Supplement: Attachment — Submitted filename: Response (2nd revision).docx [file pone.0237890.s009.docx]

Academic Editor

The authors claim to have adjusted P values as requested in the Methods, but have not done so in the Abstract, Results, Tables or Discussion, which are essentially unchanged.  It is possible to list the unadjusted P values and data, but the adjusted data needs to be included as the main findings, along with the implications of these findings and the minimal numbers of associations found after adjustment. They should also state that a future larger study would be needed to more fully assess these associations.

We all appreciate critical suggestion. As suggested, we added and modified the following sentences.

Page 3

“The allele frequencies of *DRB1*01:01*, *DRB1*04:10*, and *DRB1*15:02* were significantly higher in the IBM group than in the healthy control group (Corrected P=0.00078, 0.00038 and 0.0046).”

“There was a weak association between the *DRB1*01:01* allele and severe leg muscle weakness and muscle atrophy.”

Page 6

“Bonferroni-corrected P (corrected P) values were obtained by multiplying the observed p values by the number of DRB1 alleles (× 29).”

Page 9

“*DRB1*01:01* (17% vs. 6%, P=0.000027, corrected P=0.00078), *DRB1*04:10* (8% vs. 2%, P=0.000013, corrected P=0.00038), and *DRB1*15:02* (24% vs. 12%, P=0.00016, corrected P=0.0046) were detected in the Japanese IBM patients at higher rates than in healthy controls using Bonferroni-correction.”

“Among these alleles, *DRB1*09:01* showed still significant after a Bonferroni correction for multiple comparisons (P=5.4 × 10^-9^, corrected P=1.6× 10^-7^).”

Table 2

We added the corrected P values and foot note.

Page 12

“(i) the frequencies of *DRB1*01:01*, *DRB1*04:10*, and *DRB1*15:02* were significantly higher in the Japanese patients with IBM than in healthy controls (Corrected P=0.00078, 0.00038 and 0.0046);”

Page 13

“However, we cannot find the significant association after the Bonferroni-correction. In this regard, a future larger study with more IBM patients would be needed to fully assess the association.”
